# Exosomal miR-10b-5p mediates cell communication of gastric cancer cells and fibroblasts and facilitates cell proliferation

**DOI:** 10.7150/jca.47817

**Published:** 2021-02-21

**Authors:** Ting Yan, Xiaping Wang, Guohua Wei, Hai Li, Leiyu Hao, Yan Liu, Xinqian Yu, Wei Zhu, Ping Liu, Yichao Zhu, Xin Zhou

**Affiliations:** 1 Safety Assessment and Research Center for Drug, Pesticide and Veterinary Drug of Jiangsu Province, Nanjing Medical University, Nanjing 211166, China.; 2 Department of Pathology, The Second Affiliated Hospital of Nanjing Medical University, Nanjing 210000, China.; 3 Department of Anesthesiology, First Affiliated Hospital of Nanjing Medical University, Nanjing 210029, China.; 4 Department of Pathology, First Affiliated Hospital of Nanjing Medical University, Nanjing 210029, China.; 5 Department of Physiology, Nanjing Medical University, Nanjing 211166, China.; 6 Department of Oncology, First Affiliated Hospital of Nanjing Medical University, Nanjing 210029, China.; 7 State Key Laboratory of Reproductive Medicine, Nanjing Medical University, Nanjing 211166, China.

**Keywords:** miR-10b-5p, exosome, gastric cancer, cell proliferation, fibroblast

## Abstract

Tumor microenvironment interacts with gastric cancer (GC) cells and affects tumor development. The communication between GC cells and fibroblasts has not been clearly studied and understood. MiR-10b-5p was found highly expressed in tissue and serum samples of patients with advanced stages (stage III+IV) than that in early stage patients (stage I+II). The expression determination of serum exosomal microRNA was also shown with high expression of miR-10b-5p in GC patients with advanced stages. Dual-luciferase activity assays indicated that miR-10b-5p targeted *PTEN* in GC cells and *KLF11* in fibroblasts. The silence of miR-10b-5p up-regulated the expression of PTEN and repressed PI3K/Akt/mTORC1 signaling in GC cells. Clonogenic assay and MTT assay demonstrated that miR-10b-5p inhibitor could significantly reduce the colony formation and cell viability of GC cells. And the incubation of exosomal miR-10b-5p could increase the proliferation of GC cells. Immunohistochemistry staining revealed that high expression of α-SMA was detected in GC tissues with advanced stages. The overexpression of miR-10b-5p down-regulated KLF11 expression and elevated TGFβR1 expression in fibroblasts. In addition, miR-10b-5p inhibitor blocked the secretion of TGFβ1 in GC cells and the directional migration of fibroblasts. Therefore, up-regulated exosomal miR-10b-5p is involved in the interaction of GC cells and fibroblasts in tumor microenvironment *via* participating in the regulation of TGFβ signaling pathway.

## Introduction

Gastric cancer (GC) is the fifth frequently diagnosed cancer and the third leading cause of cancer deaths all around the world in 2018 1. Despite of the advances in treatment regimens, prognosis of GC patients is still poor, mainly due to metastasis or recurrence, resulting in a low 5-year survival rate of < 20% 2-4. It is gradually recognized that malignant cells and tumor stroma act as conjunct participants and regulators to modify tumor microenvironment and regulate tumor development 5. As the most abundant types of stroma cells, fibroblasts in tumor microenvironment could facilitate the proliferative and survival propensities of cancer cells and favor tumorigenesis in most solid tumors, including GC 6-8. Thus, further studies on the interaction between GC cells and fibroblasts could provide an insight into the underlying mechanism, and improve identification of potential treatment targets for GC.

MicroRNAs (miRNAs), approximately 19-22 nucleotides in length, are a class of highly conserved RNAs that function as post-transcriptional regulators by inducing mRNA degradation or translational inhibition 9, 10. For the first time, the role of miR-10b-5p targeting BDNF is reported in Huntington's disease *via in silico* analysis 11. The dysregulation of miR-10b-5p in Huntington's disease brain relates to age of onset and the extent of striatal involvement 12. In the study of cancer, the overexpression of miR-10b-5p suppresses the expression of CREB1 and inhibits cell proliferation, migration and apoptosis reduction of renal cell carcinoma 13. Bioinformatics analysis demonstrates that miR-10b-5p is down-regulated in breast cancer and low expression of miR-10b-5p is significantly correlated to worse outcomes 14. Long intergenic non-protein coding RNA 324 (LINC00324) is mainly distributed in the cytoplasm, fostering the expression of E-cadherin by sponging miR-10b-5p 15. The circ_0021977/miR-10b-5p/p21 and p53 regulatory axis suppresses proliferation, migration, and invasion of colorectal cancer cells 16. Serum exosomal microRNA miR-10b-5p is a potential biomarker for early-stage hepatocellular carcinoma 17. However, the role of serum exosomal miR-10b-5p has not been illuminated in GC.

In this study, we determined the expression of miR-10b-5p in GC tissue samples, serum samples, and exosomes samples derived from serum. We demonstrated that miR-10b-5p targets *PTEN* of gastric cancer cells and *KLF11* of fibroblasts and mediates the communication of gastric cancer cells and fibroblasts *via* TGFβ1/TGFβR1 signaling.

## Materials and methods

### Patients and samples

In the study, 196 tissue samples (169 GC samples and 27 normal gastric mucosa samples), 323 serum samples (145 GC samples and 178 normal controls) and additional 121 exosomes samples derived from serum (69 GC samples and 52 normal controls) were obtained from the First Affiliated Hospital of Nanjing Medical University from 2013 to 2016. All patients provided the written informed consent and did not accept any therapeutic intervention before this study. The GC patients' specimens were classified according to the 8th Edition of the AJCC gastric cancer staging system. The study was approved by Institutional Review Boards of the First Affiliated Hospital of Nanjing Medical University.

### Isolation of exosomes

Exosomes from serum samples were extracted using ExoQuick Exosome Precipitation Solution (System Biosciences, Mountain View, CA, USA) in accordance with the instructions. Briefly, 200 μL serum was incubated with 50 μL ExoQuick Exosome Precipitation solutions for 30-60 min at 2-8 °C followed by centrifugation at 13,000 rpm for 2 min. Then the deposit was obtained and dissolved in 200 μL RNase-free water for further RNA extraction.

### Transmission electron microscopy (TEM)

The exosomes sample diluted with PBS was spotted onto a glow-discharged copper grid for 10 min at room temperature. Then, exosomes were stained with a drop of 2% phosphotungstic acid for 2 min and subjected to be evaluated by transmission electron microscope (JEM-1010 microscope, Japan).

### RNA extraction

For tissue samples, Trizol (Invitrogen, Carlsbad, CA, USA) was used to isolate total RNA in consistent with the manufacturer's protocol. Total RNA from serum or exosomes was extracted with the mirVana PARIS Kit (Ambion, Austin, TX, USA) according to the instructions. After the addition of denaturing solution (Ambion, Austin, TX, USA), 5 μL synthetic *C. elegans* miR-39 (5 nmol/L, RiboBio, Guangzhou, China) was added into each sample for normalization of the sample-to-sample variation. Total RNA was lysed in 100 μL RNase-free water and then stored at -80 °C for further analysis.

### Quantitative real-time polymerase chain reaction (qRT-PCR)

Specific primers of reverse transcription (RT) and polymerase chain reaction (PCR) of miR-10b-5p were purchased from RiboBio Company (Guangzhou, China) and applied in the process of miRNA amplification. The process of RT and PCR were performed according to the protocol of manufacturer. MiRNAs were amplified and then detected on LightCycler^®^ 480 Real-Time PCR System (Roche Diagnostics, Mannheim, Germany), using SYBR Green dye (TaKaRa, Dalian, China). The expression of miRNAs was determined using 2^-ΔΔCt^ method relative to RNU6B (U6) for tissue samples and cel-miR-39 for serum and exosomes samples.

### Cell culture

SGC-7901 and MGC-803 human gastric cancer cell line, HEK-293T cell line, and human skin fibroblast HSF cell line were purchased from the Cell Bank of Shanghai (Shanghai, China) and cultured in Dulbecco minimum essential medium (DMEM), supplemented with 1% penicillin/streptomycin and 10% fetal bovine serum (Hyclone, Logan, UT, USA), at 37 °C in a humidified atmosphere with 5% CO_2_.

### Dual-luciferase activity assay

The 3'-UTRs of human PTEN and KLF11 containing the putative target site for the miR-10b-5p were chemically synthesized and inserted at the *Xba*I site, the downstream of the luciferase gene in the pGL3-control vector (Promega, Madison, WI), respectively. HEK-293T, SGC-7901, and HSF cells were plated in 24-well plates with a density of 1.5×10^5^ cells/well 24 h before transfection. Two hundred ng of pGL3-PTEN-3'-UTR or pGL3-KLF11-3'-UTR plus 80 ng pRL-TK (Promega) were transfected in combination with 60 pmol of the miR-10b-5p mimic or miRNA mimic control using Lipofectamine 2000 (Invitrogen) according to the manufacturer's protocol. Luciferase activity was measured 24 h after transfection using the Dual Luciferase Reporter Assay System (Promega). Firefly luciferase activity was normalized to renilla luciferase activity for each transfected well.

### Western blot analysis

SGC-7901 or HSF cells were plated in 6-well plates with a density of 6×10^5^ cells /well. Cells were harvested and homogenized with lysis buffer72 h after the transfection of miR-10b-5p inhibitor or miR-10b-5p mimic. Total proteins were separated by 10% SDS-polyacrylamide gel electrophoresis. Western blot analysis was performed as the usual protocol. The primary antibodies for PTEN, Phospho-S6, Phospho-Akt, KLF11, TGFβR1 and GAPDH were purchased from Abcam (Cambridge, MA, USA). Protein levels were normalized to GAPDH.

### Clonogenic assay

SGC-7901 or MGC-803cells plated into 6-well plates with a density of 1000 cells/well were transfected with miR-10b-5p inhibitor, treated with 1 μmol/L Oroxin B (Selleck, Houston, TX, USA), treated with 2 μmol/L SF1670 (Selleck), or cultured with the supernatant of MGC-803 cells' culture medium. After 10 days culture, cells were fixed and stained with crystal violet. The number of colonies were counted under a microscope from three independent replicates.

### MTT (cell viability) assay

The 3-(4,5-dimethylthiazol-2-yl)-2,5-diphenyltetrazolium bromide (MTT) assay was used for the assessment of cell viability. SGC-7901 or MGC-803 cells were seeded on 96-well plates in 100 mL medium for each well, cultured for 24 h, and then were made quiescent by serum starvation for 24 h. After 24 h culture, 20 μL MTT solution was added to each well followed by incubation for 4 h. After removal of the medium, 150 μL dimethylsulfoxide (DMSO) was added to each well. After gentle shaking, absorbance at 490 nm was measured. All absorbance value was normalized to that in a control value.

### Immunohistochemistry staining

The primary anti α-SMA antibody (Cell Signaling Technology, Danvers, MA, USA) was used. Antibody staining was visualized with DAB and hematoxylin counterstain (ZSGB-Bio, China). The percentage of positively stained cells was scored on a scale of 0 to 3 as follows: 0: <1%, 1: 1-30%, 2: 31-75%, and 3: >75%. The staining intensity was scored from 0 to 1 as follows: 0: negative, 0.5: weak, and 1: strong. The immunoreactivity score (IRS) for each case was generated by multipling percentages of positive cells and staining intensities. Immunostained sections were photographed using a microscope (Olympus Corporation, Tokyo, Japan).

### ELISA

SGC-7901 and MGC-803cells transfected with miR-10b-5p inhibitor or miR-Ctrl were cultured for 48 h. The supernatants of SGC-7901 and MGC-803cells' culture medium were collected and subjected to TGFβ1 ELISA assays (Multi Sciences, Hangzhou, China). The process of ELISA assay was performed according to the protocol of manufacturer. In the end, the absorbance of each well was read at 450 nm.

### Cell migration assay

Cell migration was assessed in a modified Boyden chamber system (Costar, Corning, NY, USA), in which the two chambers were separated by a polycarbonate membrane (8.0-μm pore diameter). SGC-7901 cells transfected with miR-10b-5p or treated with 50 nmol/L Galunisertib (LY2157299, Selleck, Houston, TX, USA) were plated on the top of Boyden chambers. The cells were allowed to migrate for 6 h. Thereafter, the stationary cells were removed with a cotton-tipped applicator, and the membranes were cut off of the chamber and stained with 0.2% crystal violet. The number of migratory cells was counted under an optical microscope.

### Statistical analysis

Mann-Whitney test was conducted to assess the difference of expression level of miR-10b-5p in different groups. All statistical analyses and graph plotting were performed using SPSS 20.0 software (SPSS Inc., Chicago, IL, USA) and GraphPad Prism 7.0 (GraphPad Software, USA). *P* value <0.05 is considered as a significant difference.

## Results

### miR-10b-5p is up-regulated in GC and associated with advanced TNM stages

**Figure 1:**
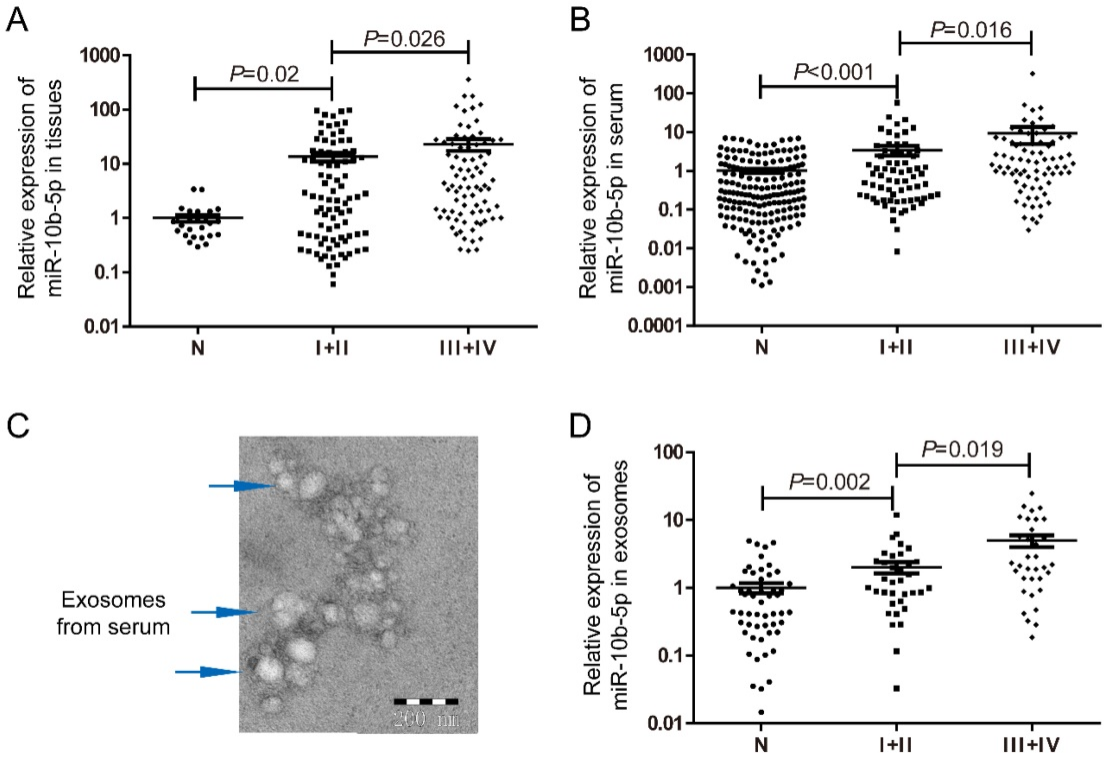
** Expression levels of miR-10b-5p in GC samples of tissue, serum and exosomes.** (A) Compared to 27 normal gastric mucosa tissue samples, miR-10b-5p was up-regulated in 87 tissue samples of GC patients with early stages and showed higher expression in 82 tissue samples with advanced stages. (B) Serum miR-10b-5p was elevated in 145 GC patients and correlated with advanced stages (n=76) compared to 179 normal controls. (C) Confirmation of exosomes extracted from serum samples with TEM to image exosomal morphology. (D) Up-regulated exosomal miR-10b-5p in GC was higher in 34 patients with advanced stages than 35 with early stages.

To determine the expression level of miR-10b-5p in GC, tissue and serum samples were retrieved in this study. According to the 8th Edition of the AJCC gastric cancer staging system, 169 tissue samples were classified as 87 patients with early stage (stage I+II) and 82 with advanced stages (stage III+IV). Compared to the normal gastric mucosa tissues, miR-10b-5p was significantly up-regulated in tissues of GC, and showed higher expression levels in patients with advanced stages (Figure 1A). Higher expression of miR-10b-5p in serum of GC was also found in 76 patients with advanced stages than that in 69 patients with early stages (Figure 1B). In addition, exosomes samples extracted from 35 patients with early stages and 34 with advanced stages as well as 52 normal controls were confirmed with TEM (Figure 1C). Similarly, expression of exosomal miR-10b-5p was increased in GC compared with NCs, and demonstrated higher expression levels in patients with advanced stages (Figure 1D).

### miR-10b-5p targets *PTEN* and mediates cell proliferation of GC

**Figure 2:**
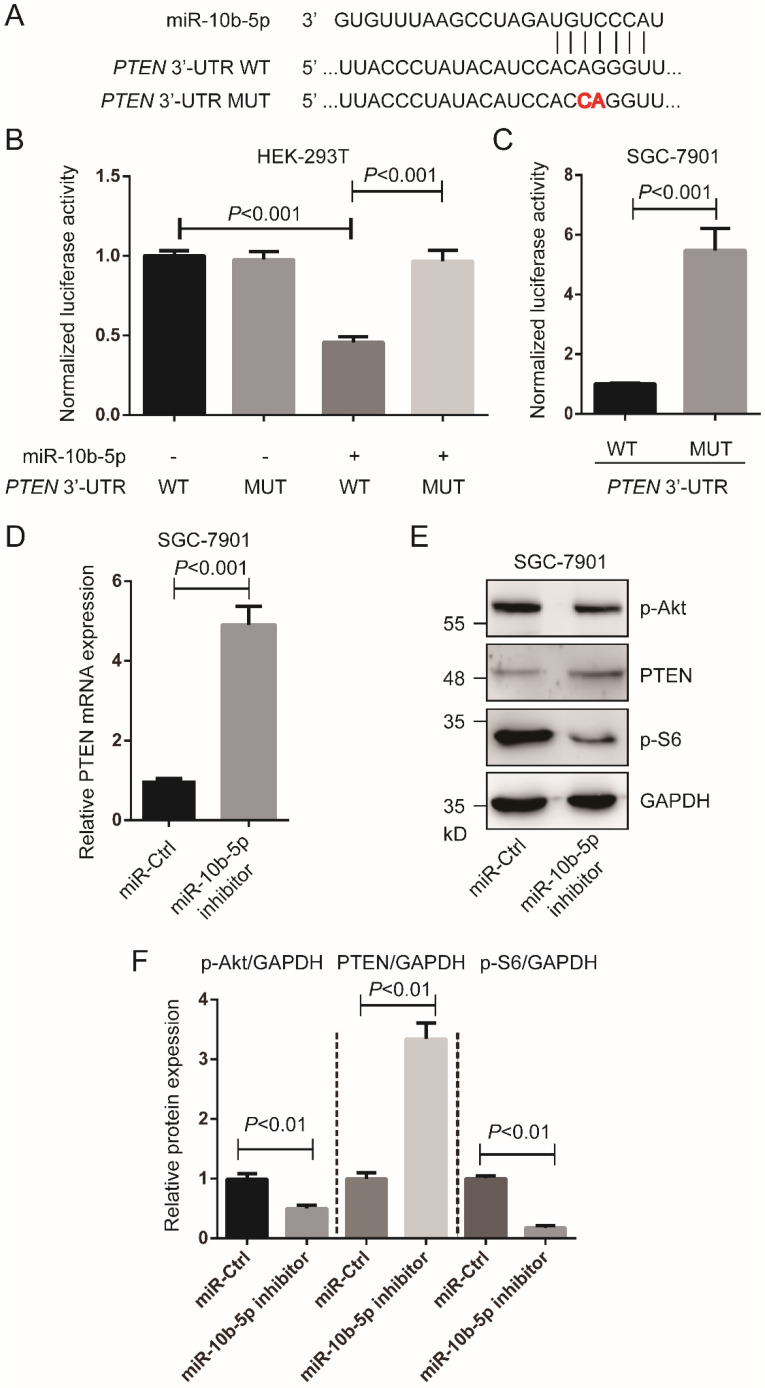
** miR-10b-5p targets *PTEN* of GC cells.** (A) The seed sequence of miR-10b-5p is complementary to the 3'-UTR of *PTEN*. (B) Luciferase assays show the reporter activity after co-transfection of wild type *PTEN* 3'-UTR (*PTEN* 3'-UTR-WT) or the mutant *PTEN* 3'-UTR (*PTEN* 3'-UTR-MUT) with miR-10b-5p into HEK-293T cells. (C) Luciferase assays show the reporter activity after the transfection of *PTEN* 3'-UTR-WT or *PTEN* 3'-UTR-MUT into SGC-7901 GC cells. (D) The mRNA expression of *PTEN* in SGC-7901 cells transfected with miR-Ctrl or miR-10b-5p inhibitor is determined by real-time PCR. U6 is the internal control. (E, F) The expression of PTEN and the phosphorylation of Akt (T308) and S6 in SGC-7901 cells transfected with miR-Ctrl or miR-10b-5p inhibitor are determined by Western blotting. GAPDH is the internal control.

According to TargetScan 7.2 (http://www.targetscan.org/vert_72/), we found* PTEN* was a target of miR-10b-5p (Figure 2A). To validate the interaction of miR-10b-5p and *PTEN*, luciferase assays were performed in HEK-293T cells and SGC-7901 GC cells. Cells transfected with wild type *PTEN* 3'-UTR and miR-10b-5p were shown significantly reduced luciferase activity compared to cells transfected with mutant *PTEN* 3'-UTR and miR-10b-5p (Figure 2B, 2C). In the following, we down-regulated the expression of miR-10b-5p with miR-10b-5p inhibitor and then measured PTEN expression (Figure 2D, 2E, 2F). The transcription and translation of PTEN were restored after the transfection of miR-10b-5p inhibitor in SGC-7901 cells (Figure 2D, 2E, 2F). Moreover, it was found that the phosphorylation of Akt (T308) and S6 (the target of mTORC1 signaling) were repressed by the silence of miR-10b-5p in SGC-7901 cells (Figure 2D, 2E, 2F).

**Figure 3:**
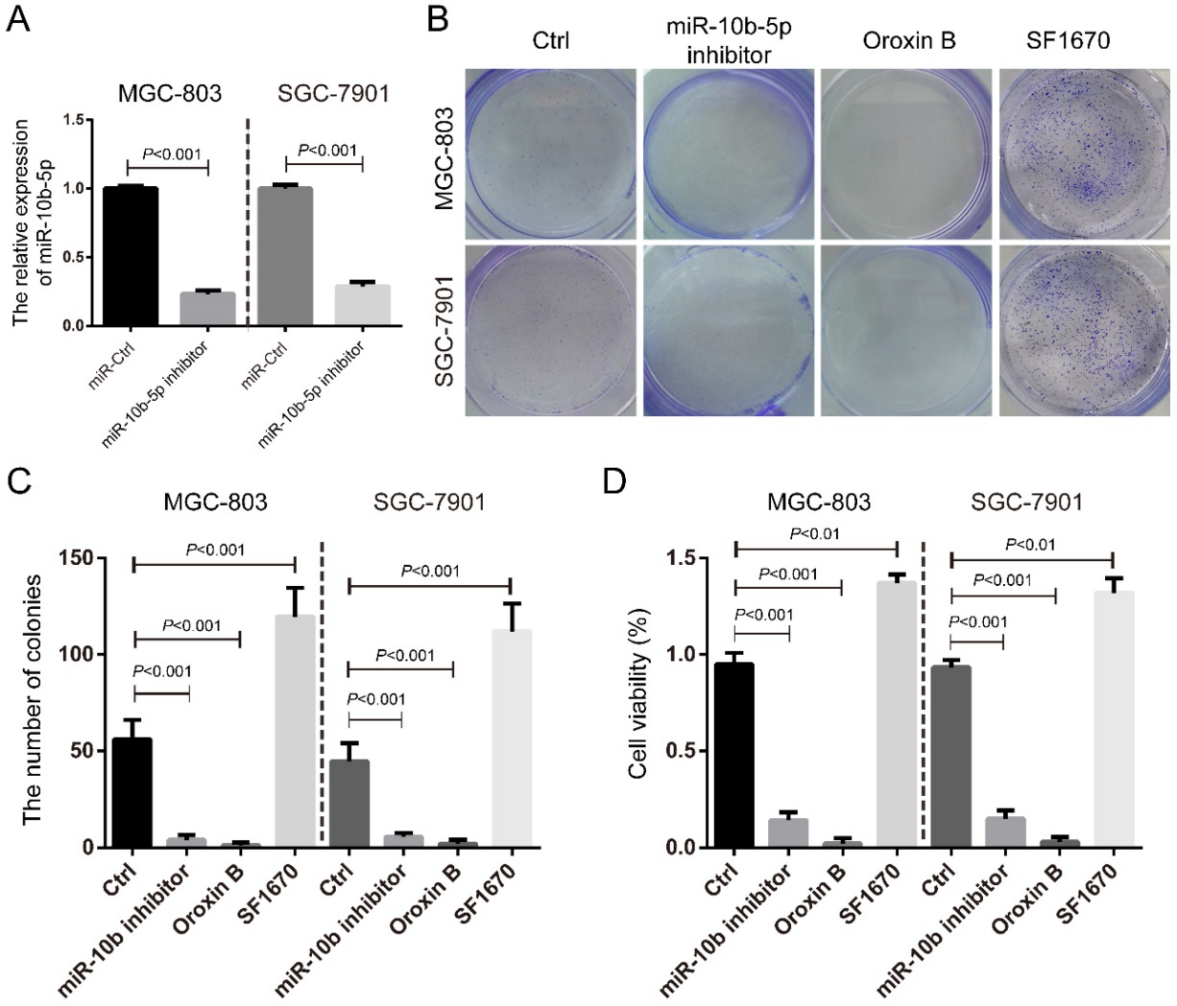
** The silence of miR-10b-5p inhibits cell proliferation of GC.** (A) The expression of miR-10b-5p in gastric cancer MGC-803 and SGC-7901 cells are blocked by the overexpression of miR-10b-5p inhibitor and determined by real-time PCR. (B, C) MGC-803 and SGC-7901 cells transfected with miR-10b-5p inhibitor, 1 µmol/L Oroxin B (the activator of PTEN) or treated with 2 µmol/L SF1670 (the inhibitor of PTEN) are subjected to colony formation assays. (D) MGC-803 and SGC-7901 cells transfected with miR-10b-5p inhibitor, 1 µmol/L Oroxin B or treated with 2 µmol/L SF1670 are subjected to MTT assays.

We further silenced miR-10b-5p by the transfection of miR-10b-5p inhibitor in MGC-803 and SGC-7901 GC cells (Figure 3A) and determined its role in cell proliferation. It was shown that miR-10b-5p inhibitor could significantly inhibit the colony formation and cell viability of GC cells (Figure 3B, 3C, 3D). The treatment of Oroxin B, the activator of PTEN, also significantly repressed the colony formation and cell viability (Figure 3B, 3C, 3D). On the contrary, the treatment of SF1670, the inhibitor of PTEN, significantly increased the colony formation and cell viability (Figure 3B, 3C, 3D). Thus, these results indicated that miR-10b-5p targets *PTEN* and elevates mTORC1 signaling, then promotes cell proliferation of GC.

### Exosomal miR-10b-5p induces the proliferation of GC cells

**Figure 4:**
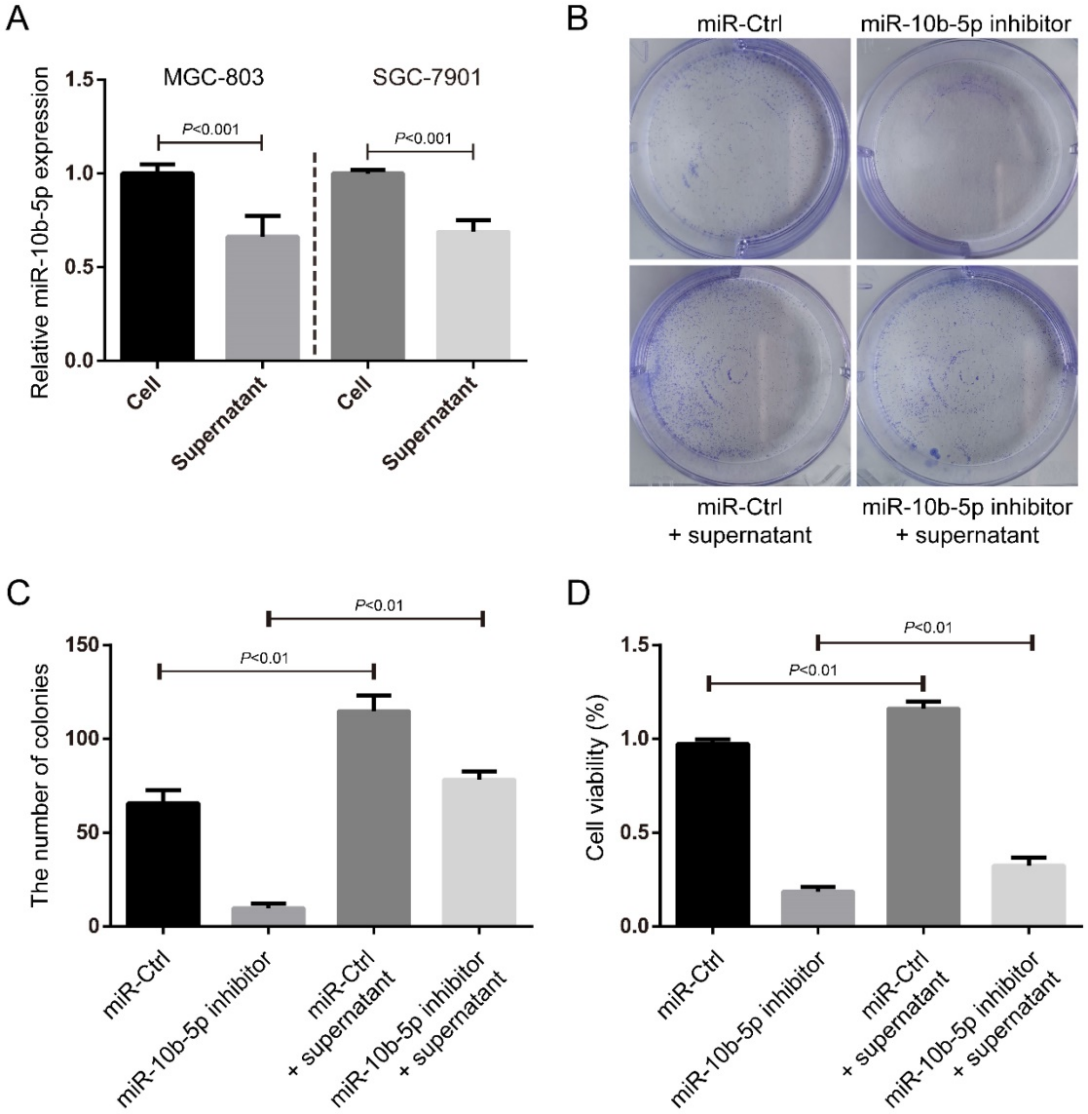
** The secretory miR-10b-5p induces cell proliferation of GC.** (A) The expression of miR-10b-5p in MGC-803 and SGC-7901 GC cells and in the supernatant of culture medium determined by real-time PCR. (B, C) SGC-7901 cells transfected with miR-10b-5p inhibitor and/or cultured with the supernatant of MGC-803 cells' culture medium are subjected to colony formation assays. (D) SGC-7901 cells transfected with miR-10b-5p inhibitor and/or cultured with the supernatant of MGC-803 cells' culture medium being subjected to MTT assays.

In the fact of that miR-10b-5p is highly expressed in exosomes of serum of GC patients with advanced stages, we hypothesized that miR-10b-5p induced a serious deterioration of GC *via* an exosome-dependent manner. We collected the supernatants of culture medium for GC cells and measured the expression of miR-10b-5p (Figure 4A). The expression of secretory miR-10b-5p was ~0.7-fold of the expression of miR-10b-5p in GC cells (Figure 4A). Next, we treated SGC-7901 cells with the supernatant of MGC-803 cells' culture medium and determined the cell proliferation rate. The condition medium largely increased the colony formation and cell viability of SGC-7901 cells (Figure 4B, 4C, 4D). Moreover, the silence of endogenous miR-10b-5p in SGC-7901 cells was unable to block the effect of exosomal miR-10b-5p secreted from MGC-803 cells (Figure 4B, 4C, 4D). These results suggested that exosomal miR-10b-5p secreted from highly malignant GC tissues aggravates the lowly malignant tissues and induces its cell proliferation.

### miR-10b-5p targets *KLF11* and up-regulates TGFβR1 of fibroblasts

**Figure 5:**
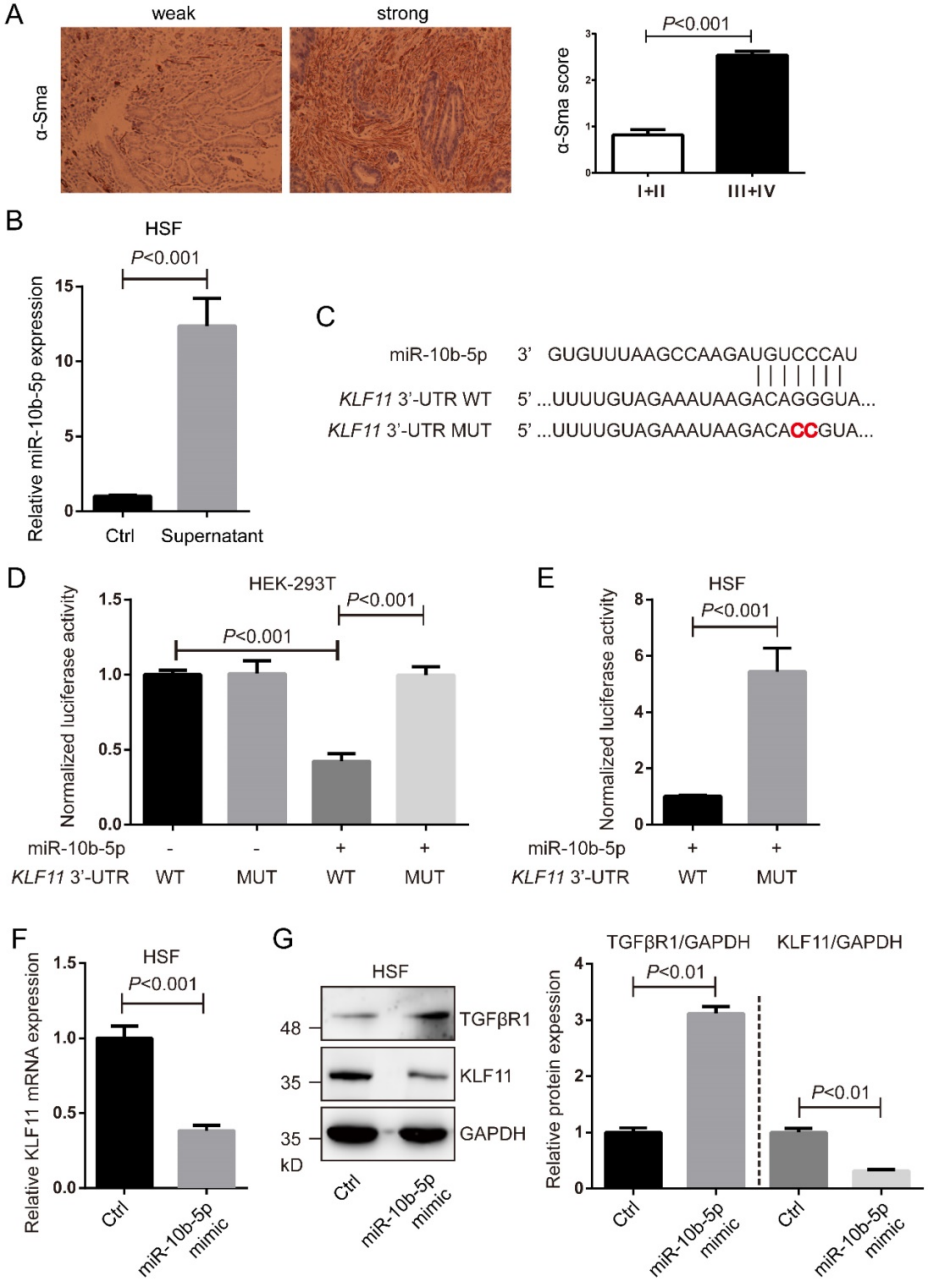
** miR-10b-5p targets *KLF11* and up-regulates TGFβR1 of fibroblasts.** (A) The representative immumohistochemical staining of α-SMA in GC tissues. The score is calculated as intensity of the staining reaction multiplied by the percentage of positive cells with a highest score of 3. Stage I+II, n=28. Stage III+IV, n=118. (B) The expression of miR-10b-5p in fibroblast HSF cells cultured with the supernatant of MGC-803 cells' culture medium is determined by real-time PCR. (C) The seed sequence of miR-10b-5p is complementary to the 3'-UTR of *KLF11*. (D) Luciferase assays show the reporter activity after co-transfection of wild type *KLF11* 3'-UTR (*KLF11* 3'-UTR-WT) or the mutant *KLF11* 3'-UTR (*KLF11* 3'-UTR-MUT) with miR-10b-5p into HEK-293T cells. (E) Luciferase assays show the reporter activity after the transfection of *KLF11* 3'-UTR-WT or *KLF11* 3'-UTR-MUT with miR-10b-5p into HSF fibroblasts. (F) The mRNA expression of *KLF11* in HSF cells transfected with Ctrl or miR-10b-5p mimic is determined by real-time PCR. U6 is the internal control. (G) The expression of KLF11 and TGFβR1 in HSF cells transfected with Ctrl or miR-10b-5p mimic are determined by Western blotting. GAPDH is the internal control.

The expression of α-SMA in GC tissues was determined by immumohistochemical staining. Higher expression of α-SMA was shown in GC tissues with advanced stages (stage III+IV, n=118) than that in GC tissues with early stages (stage I+II, n=28) (Figure 5A). Fibroblast HSF cells were cultured with the supernatant of MGC-803 cells' culture medium or normal culture medium and then determined the expression of miR-10b-5pby real-time PCR. The condition medium significantly increased the expression of miR-10b-5p, indicating that exosomal miR-10b-5p secreted from GC cells was internalized into fibroblasts (Figure 5B).

According to TargetScan 7.2, we found *KLF11* was a target of miR-10b-5p (Figure 5C). To verify the interaction of miR-10b-5p and *KLF11*, luciferase assays were performed in HEK-293T cells and fibroblast HSF cells. Cells transfected with wildtype *KLF11* 3'-UTR and miR-10b-5p were shown largely reduced luciferase activity compared to cells transfected with mutant *KLF11* 3'-UTR and miR-10b-5p (Figure 5D, 5E). Next, we over-expressed miR-10b-5p by the transfection of miR-10b-5p mimic and then measure KLF11 expression (Figure 5F, 5G). The transcription and translation of KLF11 were significantly decreased by the overexpression of miR-10b-5p mimic in HSF cells (Figure 5F, 5G). Moreover, we found that the expression of TGFβR1 was elevated by the overexpression of miR-10b-5p in HSF cells (Figure 5G).

### TGFβ1 secreted from GC promotes the migration of fibroblasts

**Figure 6:**
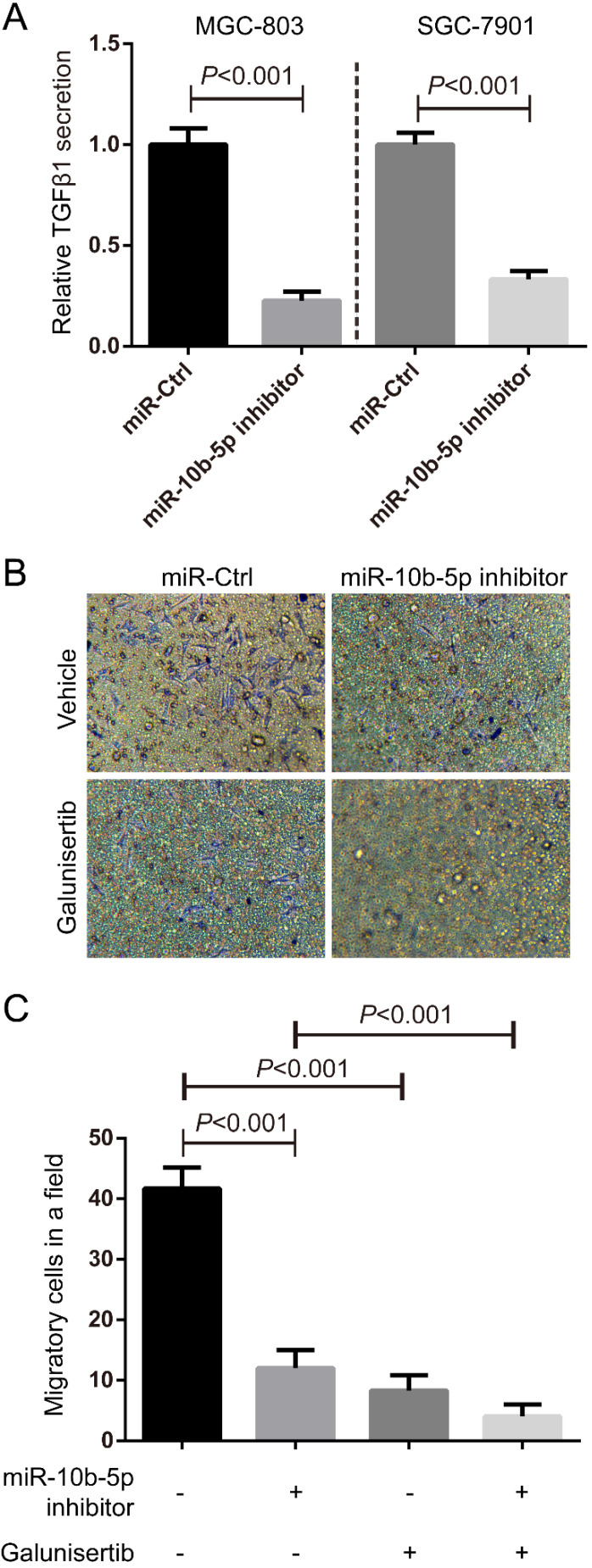
** TGFβ1 secreted from GC promotes the migration of fibroblasts.** (A) The secretory TGFβ1 of MGC-803 and SGC-7901 GC cells is blocked by the overexpression of miR-10b-5p inhibitor and determined by ELISA assays. (B, C) Fibroblast HSF cells treated with 50 nmol/L Galunisertib (the inhibitor of TGFβR1) or vehicle seeded on the top of Boyden chamber (8.0 µm pore). MGC-803 cells transfected with miR-10b-5p inhibitor or Ctrl are seeded on the bottom of Boyden chamber (8.0 µm pore). HSF cells are allowed to migrate for 6 h. The migratory HSF cells are stained with crystal violet and counted the number under a microscope. The Magnification is 100×.

**Figure 7:**
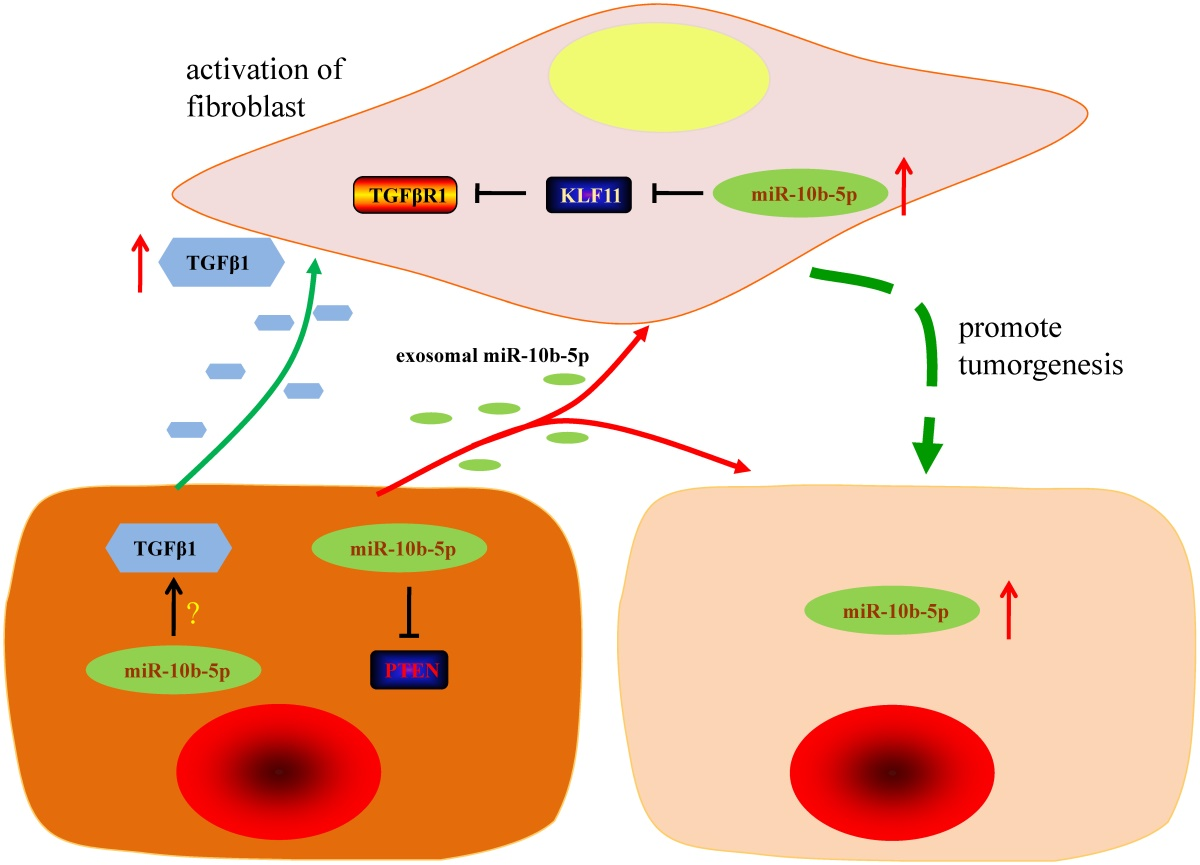
** The graphic summary of this study.** The silence of miR-10b-5p up-regulates the expression of PTEN and blocks the secretion of TGFβ1 in GC cells. Exosomal miR-10b-5p increased the proliferation of GC cells. The overexpression of miR-10b-5p down-regulates KLF11 expression and elevates TGFβR1 expression in fibroblasts. The up-regulated exosomal miR-10b-5p is involved in the interaction of GC cells and fibroblasts in tumor microenvironment *via* participating in the regulation of TGFβ signaling pathway.

In the view of the high expression of TGFβR1 in fibroblasts, we hypothesized that GC cells communicated fibroblasts *via* TGFβ1/TGFβR1 interaction. ELISA assays showed that the secretory TGFβ1 of MGC-803 and SGC-7901 GC cells was blocked by the suppression of miR-10b-5p (Figure 6A). Migratory HSF cells induced by MGC-803 cells transfected with miR-10b-5p inhibitor were less than that induced by MGC-803 cells transfected with miR-Ctrl (Figure 6B, 6C). Moreover, the treatment of Galunisertib (the inhibitor of TGFβR1) significantly inhibited the migration of fibroblasts (Figure 6B, 6C). Therefore, GC cells communicate with paracancerous fibroblasts* via* TGFβ1 signaling which can be blocked by the silence of miR-10b-5p in GC cells (Figure 7).

## Discussion

Increasing evidences demonstrate that circulating miRNAs have been stably detected in peripheral blood and may aid in detection and predicating clinical outcomes of various types of cancers 18, 19. With the protection of exosomes, exosomal miRNAs released from tumor cells are resistant against RNase digestion in circulation and more closely associated with tumor than bulk circulating miRNAs 20, 21. In addition, exosomal miRNAs secreted from tumor cells are absorbed by stroma cells, acting as important regulators among certain types of cells in tumor microenvironment 22. For example, absorption of exosomal miR-9 secreted by breast cancer cells induces the switch of normal fibroblasts to cancer-associated fibroblast-like phenotype, thus contributing to tumor growth 23. In hepatocellular carcinoma, high expression of exosomal miRNA-21 mediates intercellular crosstalk between tumor cells and hepatic stellate cells. However, few studies have explored the prominent role of exosomal miRNAs in GC 24.

In present study, we found that miR-10b-5p was highly expressed in both GC tissues and serum of GC patients with advanced stages. After the isolation of exosomes from GC patients' serum, exosomal miR-10b-5p also showed a high expression level in GC patients with advanced stages. These results indicated that serum and/or serum exosomal miR-10b-5p served as a potential diagnostic marker and was closely related to the progression of GC. The consistently up-regulated miR-10b-5p in GC samples of tissue, serum and exosomes indicates its vital role in the communication between GC cells and stroma cells in the tumor microenvironment.

In GC cells, we found up-regulated miR-10b-5p could directly target *PTEN* and regulate PI3K/Akt/mTORC1 signaling. In the study of post-myocardial infarction cardiomyocyte apoptosis, miR-10b-5p is also found to target *PTEN* and made response to hypoxic conditions in a HIF-1α-dependent manner 25. Moreover, miR-10b functions as a feedback loop to suppress IL-17A by targeting *MAP3K7* in ankylosing spondylitis Th17 cells 26. MiR-10b-5p targets *NFAT5* of C2C12 myoblasts and regulates its proliferation and differentiation 27. MiR-10b-5p targets *Apol6* of 3T3-L1 pre-adipocytes and mediates its differentiation 28. Although specific miRNA has 10~20 target genes in theory, the preponderant miRNA/mRNA axis may exist in a special pathologic niche. Here, we demonstrated the silence of miR-10b-5p up-regulates the expression of PTEN and inhibits PI3K/Akt/mTORC1 signaling, and subsequently represses the proliferation of GC cells.

In recent years, stroma cells in tumor microenvironment have received increased attention due to their participation in tumor progression, including invasion and metastasis and their ability to act as markers of clinical outcomes 29. As a major component of tumor stroma in GC, fibroblasts transfer to cancer-associated fibroblast by stimulation of TGFβ, involving in the process of tumor development 30. In our study, with staining of α-SMA antibody, we found that cancer-associated fibroblast was closely associated with advanced stage of GC patients, which was consistent with previous studies 7. Wang* et al.* found that exosomal miR-27a derived from GC cells promotes the transformation of fibroblasts into cancer-associated fibroblasts, leading to over proliferation and motility effect on GC cells 31. In present study, we applied immortalized fibroblasts cell lines, HSF, to assess the role of miR-10b-5p in the interaction of GC cells and fibroblasts. And we demonstrated that *KLF11* is a target gene of miR-10b-5p in fibroblast. As an important anti-fibrosis transcription factor, KLF11 represses the transcription of TGFβR1, resulting in the suppression of TGFβ pathway 32, 33. Consistent with previous findings, fibroblasts cultured with supernatant of GC cells' medium had shown a high expression of miR-10b-5p, which down-regulated KLF11 and up-regulated the expression of TGFβR1. Additionally, silence of miR-10b-5p inhibited the secretion of TGFβ1 in GC cells and enhanced the migratory ability of fibroblasts in presence of TGFβR1. These results demonstrate that miR-10b-5p promotes the release of TGFβ1 in GC cells and up-regulates the expression of TGFβR1 in fibroblasts, thus leading to activation of TGFβ pathway and transformation of fibroblasts to cancer-associated fibroblasts. In turn, transformed cancer-associated fibroblasts have the ability to further promote the progression of GC.

## Conclusion

Our results demonstrate that up-regulated exosomal miR-10b-5p is involved in the interaction of GC cells and fibroblasts in tumor microenvironment *via* participating in the regulation of TGFβ signaling pathway. This study provides valuable information of exosomal miRNAs as important regulators in communication of GC cells and fibroblasts, which may serve as novel treatment targets in the future.
